# A possible CPD honour system

**DOI:** 10.1177/10398562231211131

**Published:** 2023-10-31

**Authors:** Saxby Pridmore

**Affiliations:** 3925 Hobart, TAS

Dear Editor,

A psychiatrist (they, their) hoping to learn something and collect an hour of ‘development’ towards their annual CPD total, commenced an RANZCP webinar. This psychiatrist found the presentation unsatisfactory – the speakers were inaudible and the material was apparently disorganized (although the inaudibility meant the possible disorganization was uncertain). The psychiatrist left the room to get a drink, was distracted, and when they returned the Webinar had finished. The next day, the psychiatrist was offered (and downloaded) a ‘Certificate of Attendance’ (worth 60 min).

This event raises issues. One is the possibility of this psychiatrist (and potentially others) are being awarded (and later claiming) an hour of CPD credit for attending webinar to which they did not attend appropriately.

The Medical Board of Australia and the Medical Council of New Zealand have determined practitioner registration will depend on satisfactory compliance with a CPD program. The RANZCP accepted responsibility for administering such a program.^1^ Said program is complicated. Annually, 2 h must be spent on a ‘Professional Development Plan’ (which must later be reflected upon), a minimum of 10 h must be spent on ‘Peer Review Group’ activities, and a minimum of twelve and a half hours must be spent on ‘Self-Guided Learning’. A total of 50 h of CPD must be achieved (perplexing rules dictate the sections under which the additional hours can be gained).

The CPD activities of the Fellowship are quantified. On a special purpose webpage, Fellows must list the hours and under which genus they wish to claim credits. They also have the opportunity to upload substantiating documents, such as certificates of attendance at webinars. Also, designated organizers of Peer Review Groups are required to verify the attendance of individuals at group meetings. If the system detects possible problems (of if the Fellow is simply unlucky), authorities may decide to ‘audit’ a set of CPD claims – which means all must be substantiated in writing, and where possible, confirmed by witnesses.

There are more than 4000 psychiatrists between Australia and New Zealand, who must gather 50 h of CPD annually. Arguably, the size and complexity of the task make the accurate policing of CPD participation impossible. The threat to livelihood represented by deregistration as a consequence of insufficient annual CPD hours could possibly tempt/encourage Fellows to dishonesty. An alternative method would be to publicize the RANZCP expectations and replacing the current bureaucratic checking system with an honour system – giving them the opportunity to tell the truth.
